# Factors associated with positive and negative recommendations for cancer and non-cancer drugs for rare diseases in Canada

**DOI:** 10.1186/s13023-019-1104-7

**Published:** 2019-06-07

**Authors:** Fernanda Naomi Inagaki Nagase, Tania Stafinski, Jian Sun, Gian Jhangri, Devidas Menon

**Affiliations:** 1 Health Technology & Policy Unit, School of Public Health, University of Alberta, Edmonton, AB Canada; 2 Department of Medicine, University of Calgary, Calgary, AB Canada; 3 School of Public Health, University of Alberta, Edmonton, AB Canada

**Keywords:** Rare diseases, Orphan drugs, Reimbursement review

## Abstract

**Background:**

In Canada, reimbursement recommendations on drugs for common and rare diseases are overseen by the Canadian Agency for Drugs and Technologies in Health (CADTH) and made through the pan-Canadian Oncology Drug Review (pCODR) and the Common Drug Review (CDR). While the agency specifies information requirements for the review of drug submissions, how that information is used by each process to formulate final reimbursement recommendations, particularly on drugs for rare diseases (DRDs) in which per patient treatment costs are often high, is unclear. The purpose of this study was to determine which factors contribute to recommendation type for DRDs.

**Methods:**

Information was extracted from CDR and pCODR recommendations on drugs for diseases with a prevalence < 1 in 2000 from January 2012 to April 2018. Data were tabulated and multiple logistic regression was applied to explore the association between recommendation type and the following factors: condition/review process (cancer vs non-cancer), year, prevalence, clinical effectiveness (improvements in surrogate, clinical and patient reported outcomes), safety, quality of evidence (availability of comparative data, consistency between population in trial and indication, and bias), clinical need, treatment cost, and incremental cost-effective ratio (ICER). Two-way interactions were also explored.

**Results:**

A total of 103 recommendations were included. Eleven were resubmissions, all of which received a positive recommendation. Among new submissions (*n* = 92), DRDs that were safe or offered improvements in clinical or patient reported outcomes were more likely to receive positive reimbursement recommendations. No associations between recommendation type and daily treatment cost, cost-effectiveness, or condition (cancer or non-cancer) were found.

**Conclusions:**

Clinical effectiveness, as opposed to economic considerations or whether the drug is indicated for cancer or non-cancer, determine the type of reimbursement recommendation.

## Background

Coverage decision-making on new drugs for rare diseases (DRDs) challenges public and private payers, as well as the pharmaceutical industry in Canada and abroad. In addition to high per patient treatment costs, DRDs typically come with a paucity of outcomes data due in part to the small number of patients available for studies. This creates significant uncertainty around their value proposition. At the same time, DRDs often target diseases with high clinical need (i.e., they are life-threatening and/or chronically debilitating and lack active treatment/disease-modifying alternatives) [1, 2].

In Canada (unlike other countries), participating public payers rely on one of two centralised review processes (depending on disease type) to provide coverage recommendations on new DRDs. Prior to 2003, recommendations were made by individual jurisdictions (e.g., provinces and territories) through separate provincial/territorial processes or, in the case of cancer drugs, jurisdictional cancer agencies. In 2003, the centralized Common Drug Review (CDR) was launched as a means of improving the efficiency of such processes and providing high quality evidence-informed recommendations on non-cancer drugs to guide coverage decisions in all participating jurisdictional drug plans. In 2011, a parallel process, the pan-Canadian Oncology Drug Review (pCODR), was established for new cancer drugs. Both of these centralized processes are overseen by the Canadian Agency for Drugs and Technologies in Health (CADTH) [3, 4]. Although CADTH broadly specifies factors considered when formulating recommendations (clinical benefit, cost, cost-effectiveness, and patient preferences), how they are weighed relative to one another and whether it varies under certain conditions remain unclear [5].

In recent years, several analyses of centralized drug review processes have been published [6–12]. While most have focused on drugs for common conditions, three have assessed trends and factors associated with different types of coverage recommendations for DRDs. However, these studies excluded cancer drugs and examined only a small number of decision factors [6, 7, 9].

This study aimed to address the following questions:

1. Are certain factors associated with positive or negative reimbursement recommendations for drugs submitted to CDR and pCODR?
2. Does whether or not the DRD is for a cancer indication affect recommendation type?

## Methods

A quantitative analysis was conducted to address these questions. The identification and extraction of data from CDR and pCODR submissions followed guidelines for conducting systematic reviews [13].

### Identification of DRDs

All DRD recommendations from CDR and pCODR from January 2012 until April 2018 were included in the study. Before 2011, submissions for cancer drugs were reviewed by the interim Joint Oncology Drug Review (iJODR) and information on recommendations was not made publicly available. After 2011, the iJODR was formalized to a permanent body known as pCODR managed by CADTH and the first recommendation was published in 2012. Also, previous studies have shown differences in factors associated with CDR recommendations before and after the establishment of pCODR (i.e. 2012, [7, 9]. Therefore, to make recommendations comparable, CDR submissions prior to 2012 were excluded from the analyses. A rare disease was defined as a condition affecting less than 1 in 2000 people in Canada (the definition proposed in the draft pan-Canadian framework for rare diseases which had been developed by Health Canada, the national regulating body [14]). Ultra-rare diseases were defined as those with a prevalence of less than 1 in 50,000 people [15]. To identify DRDs, prevalence information was obtained from two sources: 1) the Orphanet website, an internationally governed portal for rare disease information containing a comprehensive list of rare diseases [16], and 2) a comprehensive search of published and grey literature for Canadian prevalence estimates (details of the search strategy are available from the authors).

### Collection of data on included DRDs

For each included DRD, the CDR and pCODR “Final Recommendation” documents were obtained from their respective websites [3, 4]. These documents are issued by CADTH once a reimbursement recommendation is made by an independent review committee and provide the reasons for the recommendation, considering: current available evidence on safety and clinical effectiveness, cost-effectiveness, patient input, characteristics of the drug and disease, and feasibility of adoption of the current technology (e.g. budget impact analysis and organization feasibility) [3, 4].

### Data extraction

For each included DRD, two researchers independently reviewed the relevant “Final Recommendation” document and extracted the following information using a standardized form [13]: submission type, drug name, drug type, condition/indication, final recommendation, year of final recommendation, number of randomized clinical trials (RCTs), number of patients from studies, clinical safety and efficacy/effectiveness, quality of evidence (e.g. presence of bias in outcome measures, availability of comparative data), treatment cost, and cost-effectiveness.

### Data analysis

#### Creation of variables

For each included DRD, the final recommendation was converted into a binary outcome variable coded as positive if the recommendation was to ‘list’ the drug (i.e., include it in a participating publicly funded drug benefit plan) with or without conditions and negative if the recommendation was to not ‘list’ the drug. Factors were converted to categorical variables characterizing the submissions, including the type of submission (new or resubmission), prevalence of the condition (orphan or ultra-orphan) and type of drug (alimentary tract/metabolism product, antineoplastic/immunomodulating agent or other) were created. Four binary variables (‘yes’ or ‘no/ not measured’) were created to describe the presence or absence of meaningful improvements across efficacy and effectiveness outcomes: 1) differences in clinical outcomes, 2) differences in biomarker/surrogate outcomes, and 3) differences in patient reported outcomes (PROs). Classification of the outcomes was based on the definitions described in the “Final recommendation” documents. The following binary (‘yes’ or ‘no’) variables were also created: safety issues, bias in outcome measures, consistency between the patient population in trials and indication(s) for which a reimbursement/listing recommendation was sought, availability of direct comparative data, availability of long-term data, and presence of other methodological or study design issues. A detailed description of these variables is provided in Table 1.

**Table 1:** Description of variables included in the analyses

Variable	Values	Details
Recommendation	0 if negative 1 if positive	• Negative: do not list • Positive: list, list with conditions, list with criteria, list if price reduced or cost-effectiveness improved
Submission characteristics		
Year of recommendation	Continuous variable	• Year of final recommendation
Type of submission	0 if new submission 1 if resubmission	• Type of submission according to CADTH classification
Presence of RCTs	0 if no 1 if yes	• RCTs were included in the systematic review
Therapeutic class of drugs	0 if alimentary tract & metabolism 1 if Antineoplastic & immunomodulating 2 if other	• Classification of drugs based on ATC codes
Characteristics of disease		
Type of condition	0 if cancer 1 if non-cancer	• Classification based on ICD-10
Prevalence	0 if ultra-orphan 1 if orphan	• Ultra-orphan: < 1 in 100,000 people • Orphan: < 1 in 2000 people
Clinical need	0 if no or not stated 1 if yes	• Need for alternative treatment options, no existing treatment or “unmet need”
Clinical safety/efficacy		
Safety issues	0 if yes 1 if no	• Concerns over potential serious life-threatening adverse events or unknown safety profiles
Improvements in biomarker/ surrogate outcome	0 if no, inconsistent or not measured 1 if yes	• Biomarker is “a defined characteristic that is measured as an indicator of normal biological process, pathogenic process, or responses to an exposure or intervention, including therapeutic interventions” [17]. • Surrogate outcome is “an endpoint that is used in clinical trials as a substitute for a direct measure of how a patient feels, functions, or survives” [17]. • Meaningful improvements defined as statistically significant differences or non-inferiority in biomarker/ surrogate outcomes (e.g. weight, 6 min walk test, progression-free survival)
Improvements in clinical outcomes	0 if no, inconsistent or not measured 1 if yes	• Clinical outcome is “an outcome that describes or reflects how an individual feels, functions or survives” [17]. • Meaningful improvements defined as statistically significant differences or non-inferiority in clinical outcomes (e.g. survival, transplantation)
Improvements in PRO	0 if no, inconsistent or not measured 1 if yes	• PRO is “a measurement based on a report that comes directly from the patient about the status of a patient’s health condition without amendment or interpretation of the patient’s response by a clinician or anyone else” [17]. • Meaningful improvements defined as statistically significant differences or non-inferiority in PRO (e.g. QOL, rating of pain intensity, SF-36)
Quality of evidence		
Availability of comparative data	0 if no 1 if yes	• Based on availability of direct head-to-head comparative studies (where comparators were available)
Consistency between population in trials and indications	0 if no 1 if yes	• Present when ‘final recommendation’ document stated that data from trials included all subgroup of the indicated population • Not present when for example submitted indication includes mild, moderate, and severe forms of disease but trial data limited to mild-moderate forms of disease
Bias in outcome measures	0 if yes 1 if no	• Present when indicated in the final recommendation document • Bias in outcome measurements (e.g., subjective outcomes classified by non-blinded investigators)
Long term data	0 if no 1 if yes	• Presence of long-term data where long-term data is important given the course of disease • Present when indicated in the final recommendation document
Other study design issues	0 if yes 1 if no	• Concerns over other aspects of study design (e.g., small sample size, carry-over effects associated with withdrawal trial methodology) • Present when indicated in the final recommendation document
Cost/ cost-effectiveness		
Daily treatment cost	Continuous variable in $CDN/ patient	• Average daily treatment cost of drugs per patient
ICER	Continuous variable in $CDN/QALY	• ICER calculated by CADTH or by manufacturer if no ICER calculated by CADTH was available

*ATC* Anatomical Therapeutic Chemical (ATC) Classification System, *CADTH* Canadian Agency for Drugs and Technologies in Health, *ICD* International Classification of Disease, *ICER* Incremental Cost-effectiveness Ratio, *ICU* Intensive Care Unit, *PRO* Patient- reported Outcome, *QALY* Quality-adjusted Life Years, *QOL* Quality of Life, *RCT* Randomized Controlled Trial

#### Statistical analysis

First, a series of two-by-two or three-by-two tables were constructed to examine the percentage of positive recommendations for each variable extracted from the “Final recommendation” document. Data were tabulated for all included recommendations and stratified by type of condition (i.e. cancer and non-cancer) to examine whether the frequency of positive and negative recommendations for each factor (i.e., independent variable) varied with type of condition. Pearson’s chi-square or Fisher’s exact test were used to test the statistical significance of differences in such percentages. This step was also used to check for any errors and spot complete and quasi-complete separation of data (i.e. recommendations were almost perfectly predicted by the independent variables).

Next, factors potentially associated with recommendation type were further explored through multiple logistic regression- a statistical analysis that allows for the assessment of the association between multiple factors and a dichotomous outcome (in this case, positive or negative recommendation) [18]. Two methods for building regression models were used and the results compared: 1) purposeful selection and 2) stepwise selection.

In purposeful selection, covariates whose univariate test had a *p*-value < 0.21 were first identified [18, 19]. A multivariable model containing these covariates was constructed, and variables with *p*-values > 0.21 were excluded. Each variable not selected initially for inclusion in the multivariable model was then added one at a time. If its *p*-value was > 0.05 and none of the coefficients in the model changed by > 20%, the variable was excluded. The resulting model comprised the main effects model. Finally, two-way interactions among the variables were added to the main effects model one at a time and checked for statistical significance. Those with *p*-values > 0.05 were excluded. To assess the fit of final model, the Hosmer-Lemeshow goodness-of-fit test was used [20, 21].

In stepwise selection, each variable was entered into the model step by step (SAS® Stepwise Logistic Regression). The significance level for entry and stay were set at 0.2. The results were identical with purposeful method.

## Results

**Fig. 1:**
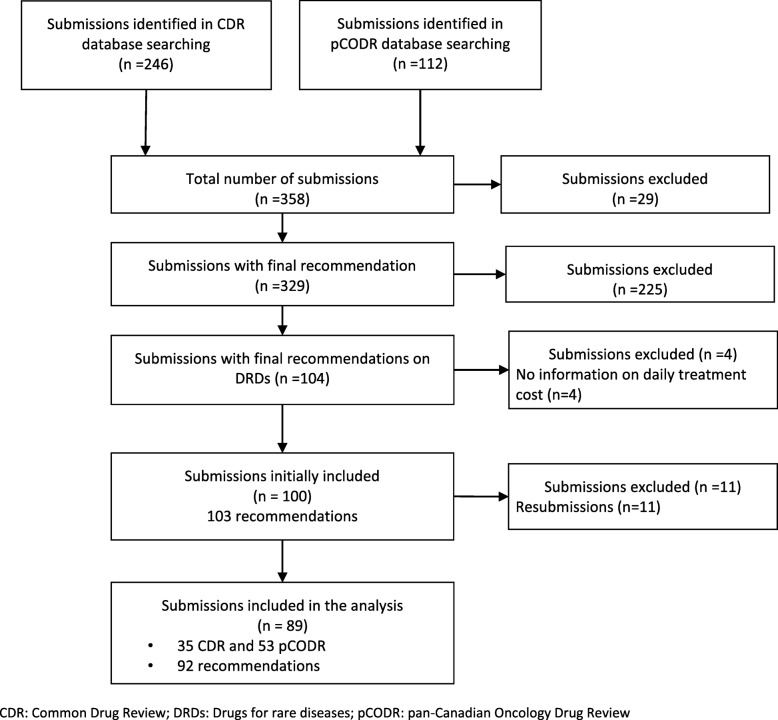
Flow diagram of the search and selection of submissions of DRDs

Initially, 104 submissions (42 CDR and 62 pCODR) with final recommendations on DRDs were identified. Fifteen were excluded (11 resubmissions and 4 with no daily treatment cost information), leaving a total of 88 submissions comprising 92 final recommendations for inclusion in the analysis (Fig. 1). Resubmissions (*n* = 11) were excluded since they all received a positive recommendation. Of the 103 recommendations, 82 (80%) were positive (Table 2). Most recommendations were for antineoplastic & immunomodulating therapies, but the proportion of positive recommendations among different ‘therapeutic class of drugs’ were similar.

**Table 2:** Overall description of included recommendations

Factors	n	Positive recommendations	% Positive	*p*-value
All	103	82	79.6	–
Therapeutic class of drug				0.711
Alimentary tract & metabolism	11	10	90.9	
Antineoplastic & immunomodulating	78	61	78.2	
Others	14	11	78.6	
Type of condition				0.879
Cancer	66	53	80.3	
Endocrine	16	12	75.0	
Others	21	17	80.9	
Type of submission				0.070
New	92	71	77.2	
Resubmission	11	11	100.0	

**Fig. 2:**
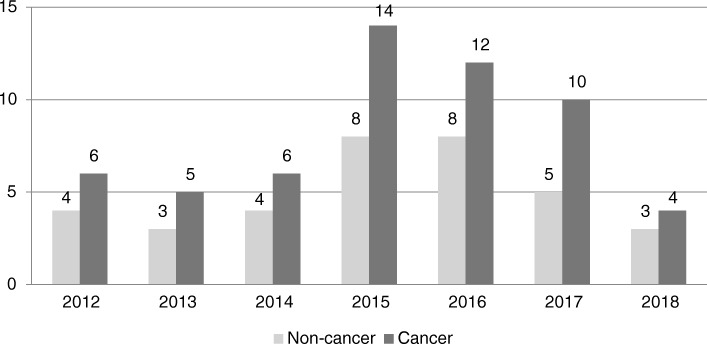
Distribution of recommendations of new submissions by year of final recommendation

**Fig. 3:**
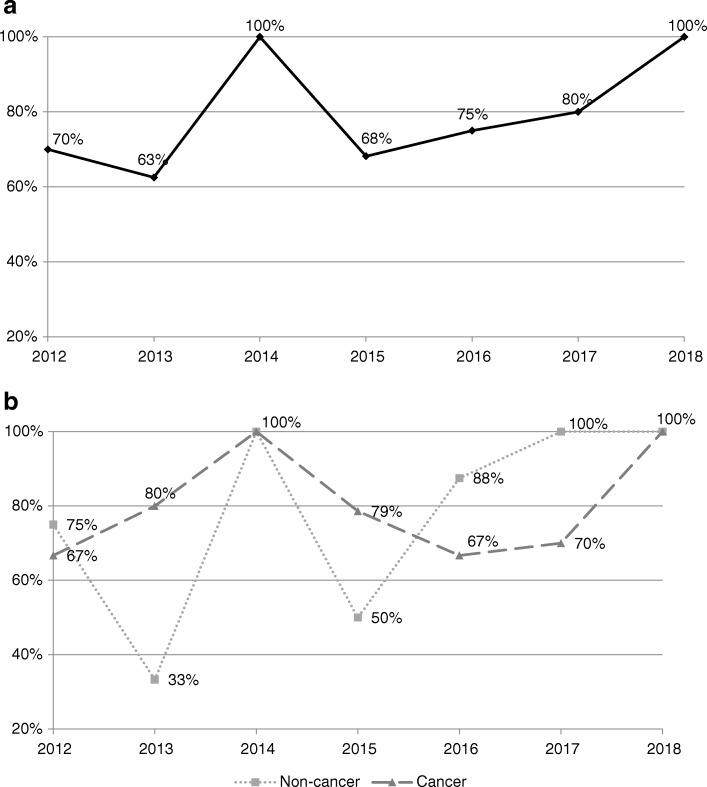
Percentage of positive recommendations by year of recommendations for: (**a**) all new submissions and (**b**) all new submissions stratified by type of condition

Figure 2 shows the number of recommendations on new submissions made each year since 2012. From 2012 to 2014, the average was around 9 per year, whereas after 2015, the average increased to 19 (2018 was excluded since the data were only available for the first quarter of the year). Overall, the proportion of positive recommendations on new submissions remained high (ranging from 63 to 100%). While the proportion of positive recommendations on cancer DRDs remained similar over the years, that for non-cancer DRDs increased after 2015 (Fig. 3). However, the number of non-cancer DRD submissions was also small prior to 2015.

Table 3 provides information on the distribution of positive recommendations according to each potential decision factor and stratified by cancer and non-cancer drugs. Twenty-five (96%) out of 26 recommendations showing ‘improvement in clinical outcomes’ were positive. Only one in cancer reporting ‘improvements in clinical outcomes’ was negative. Likewise, 24 of 25 (96%) recommendations reporting ‘improvements in PROs’ were positive and the proportion of positive recommendations was similar for cancer and non-cancer DRDs.

**Table 3:** Distribution of positive recommendations for all new submissions of DRDs

	All new submissions				Non-Cancer drugs				Cancer drugs			
Factors	n	Positive recommendations	% Positive	*p*-value*	n	Positive recommendations	% Positive	*p*-value*	n	Positive recommendations	% Positive	*p*-value*
All	92	71	77.2		35	27	77.1		57	44	77.2	
Type of condition				0.996								
Cancer	57	44	77.2									
Non-cancer	35	27	77.1									
*Submission characteristics*												
Presence of RCTs				0.083				0.033				0.727
No	22	14	63.6		7	3	42.9		15	11	73.3	
Yes	70	57	81.4		28	24	85.7		42	33	78.6	
Therapeutic class of drug				0.714				0.418		NA		
Alimentary tract & metabolism	10	9	90.0		10	9	90.0					
Antineoplastic & immunomodulating	68	51	75.0		11	7	63.6					
Others	14	11	78.6		14	11	78.6					
*Characteristics of disease*												
Prevalence				1.000				1.000				1.000
Ultra- orphan	14	11	78.6		8	6	75.0		6	5	83.3	
Orphan	78	60	96.9		27	21	77.8		51	39	76.5	
Clinical need				0.051				0.419				0.070
No/ not stated	28	18	64.3		20	14	70.0		8	4	50.0	
Yes	64	53	82.8		15	13	86.7		49	40	81.6	
*Clinical safety/ efficacy*												
Safety issues				0.021				0.226				0.102
Yes	33	21	63.6		11	7	63.6		22	14	63.6	
No	59	50	84.7		24	20	83.3		35	30	85.7	
Improvements in biomarker/surrogate outcomes				0.316				0.228				0.006
No/ inconsistent/ not measured	27	19	70.4		17	15	88.2		10	4	40.0	
Yes	65	52	80.0		18	12	66.7		47	40	85.1	
Improvements in clinical outcomes				0.005				0.073				0.084
No/ inconsistent/ not measured	66	46	69.7		25	17	68.0		41	29	70.7	
Yes	26	25	96.1		10	10	100.0		16	15	93.7	
Improvements in PRO				0.010				0.299				0.042
No/ inconsistent/ not measured	67	47	70.1		29	21	72.4		38	26	68.4	
Yes	25	24	96.0		6	6	100.0		19	18	94.7	
*Quality of evidence*												
Availability of comparative data				0.427				1.000				0.510
No	33	27	81.8		14	11	78.6		19	16	84.2	
Yes	59	44	74.6		21	16	76.2		38	28	73.7	
Consistency between population in trials and indications				0.130				0.431				0.345
No	48	34	70.8		21	15	71.4		27	19	70.4	
Yes	44	37	84.1		14	12	85.7		30	25	83.3	
Bias in outcome measures				0.503				0.216				1.000
Yes	54	43	79.6		12	11	91.7		42	32	76.2	
No	38	28	73.7		23	16	69.6		15	12	80.0	
Long term data				0.186				0.390				0.346
No	62	45	72.6		25	18	72.0		37	27	73.0	
Yes	30	26	86.7		10	9	90.0		20	17	85.0	
Other study design issues				0.202				1.000				0.044
Yes	58	42	72.4		19	15	78.9		39	27	69.2	
No	34	29	85.3		16	12	75.0		18	17	94.4	
*Cost/ cost-effectiveness*												
Daily treatment cost				1.000				0.298				0.258
≤ 150	19	15	78.9		13	12	92.3		6	3	50.0	
150–500	52	40	76.9		8	5	62.5		44	35	79.6	
> 500	21	16	76.2		14	10	71.4		7	6	85.7	
ICER in $CDN/QALYs ^a^				0.647				1.000				0.194
≤ 100,000	12	11	91.7		2	2	100.0		10	9	90.0	
100,000-500,000	48	37	77.1		7	6	85.7		41	31	75.6	
> 500,000	16	13	81.2		12	11	91.7		4	2	50.0	

*DRDs* Drugs for rare diseases, *ICER* Incremental cost-effectiveness ratio, *NA* Not applicable, *PRO* Patient-reported outcomes, *RCT* Randomized controlled trial

*p-values based on Pearson’s chi-square statistic or Fisher’s exact test

^a^Data on ICER was only available for 76 recommendations

The proportion of positive recommendations for those with no ‘safety issues’ was similar between non-cancer (83%) and cancer (86%) DRDs. However, only 40% of cancer drugs showing no evidence of ‘improvements in biomarker/ surrogate outcomes’ received a positive recommendation, versus 88% of non-cancer drugs.

Neither ‘daily treatment cost’ nor ‘ICERs’ was associated with recommendation type, but the distribution of recommendations related to these factors appeared to differ between cancer and non-cancer DRDs (Table 3).

After controlling for potential confounders through multiple logistic regressions, only three factors were identified as statistically significantly associated with recommendation type: 1) ‘Safety issues’, 2) ‘Improvement in clinical outcomes’, and 3) ‘Improvement in patient reported outcomes’ (Table 4). In all three, DRDs that did not reported ‘safety issues’ or showed significantly improved clinical outcomes or patient reported outcomes were more likely to receive a positive listing recommendation. No factors were found to be statistically significantly associated with negative recommendations.

**Table 4:** Results of multiple regression analysis of DRDs

Variables in the model	OR (95%CI)
Presence of RCTs (ref.: no)	2.9 (0.7; 11.8)
Safety issues (ref: yes)	4.0 (1.2; 13.6)
Improvements in clinical outcomes (ref: yes)	20.6 (2.2; 189.7)
Improvements in patient reported outcomes (ref: yes)	12.1 (1.3; 110.5)
Consistency between population in trial and indications (ref: no)	3.5 (0.9; 12.7)

*95%CI* 95% confidence interval, *DRDs* drugs for rare diseases, *OR* odds ratio, *RCT* randomized controlled trial, *ref*. reference

## Discussion

This study examined the potential relationship between factors considered during deliberations by centralized drug review committees in Canada and final recommendation. Cancer drugs were no more or less likely to receive a positive recommendation than those for non-cancer. Similarly, no correlation between per patient treatment cost or size of ICER and type of recommendation was found, suggesting that the economic implications of a DRD are not driving deliberations. In contrast, DRDs that offered improvements in clinical outcomes, or PROs were more likely to receive positive recommendations. Similar to our results, two previously published studies of CDR recommendations determined that clinical effectiveness was a strong predictor of recommendation type and there was no association between the size of the ICER and recommendation type [9, 10]. It may be argued that decision makers consider economic models useful when supported by strong clinical evidence [22], which is not usually available for DRDs [23]. In the case of DRDs, other societal considerations such as the principle of social solidarity and the right to health may play a more important role [23, 24].

In recent years, CADTH has made efforts to better align the CDR and pCODR processes [5, 25]. The lack of differences in recommendations based on type of indication suggests that their efforts have been successful. This now raises questions around the need for two review processes. To our knowledge, no other countries with centralized drug reviews have created separate processes for cancer drugs. While previously published studies have found no rationale for the establishment of pCODR [26], it has been argued that while unclear, there may be good reasons [27].

Overall, the proportions of positive recommendations on new submissions were high, although they fluctuated between 2012 and 2015. From 2012 to 2018, two changes in the deliberative framework may have contributed to the increase in positive recommendations. In November 2012, CADTH published a framework for CDR in which price reduction was added as a condition for listing the drug. The framework also included a category of “do not list at submitted price”, which before 2012, was a subcategory of the “do not list” category [28]. In March 2016, the wording of recommendations was modified once again and the categories of recommendations were reduced to three: “reimburse”, “reimburse with clinical criteria and/ or conditions” and “do not reimburse”. A negative recommendation around price no longer appears to exist, and costly drugs or those with unfavourable ICERs can receive a recommendation under the category of “reimburse conditional of reduced price”. Further, this framework provides the option of issuing a positive recommendation in “exceptional cases” in which there are uncertainties around the effectiveness of a drug. “Exceptional cases” may be relevant to rare diseases (i.e., the drug is for life-threatening conditions and/ or affects a small population) [5]. While our data show an increase in positive recommendations since 2016, further research with long-term data is required in order to investigate the impact of these changes in a more robust way.

### Limitations

This study has five main limitations. First, for some recommendations, a judgement call was required in order to classify them as positive or negative. For example, “list if … substantial reduction in price” may be considered a negative recommendation because it could ultimately yield a negative reimbursement decision. However, similar to previously published work, we considered a positive recommendation to be one in which manufacturers were able to proceed to the next stage, which was price negotiation and reimbursement [29]. Second, the sample size was small and information on some of the variables was not available. Third, the association between feasibility, one of the factors described in documents emerging from pCODR deliberations, and recommendation type was not evaluated, since similar information was not available for drugs reviewed by the CDR. Nonetheless, adoption feasibility takes into account budget impact, which may be an important consideration during pricing and reimbursement decision-making [30]. Fourth, biomarker and surrogate outcomes were included in the same category due to small sample size. According to the FDA, surrogate outcomes are biomarkers that can predict clinical outcomes [17, 31]. In the case of DRDs, particularly those for non-cancer indications, long-term studies designed to gather information on the natural progression of the disease are lacking. Consequently, there is a reliance on biomarkers as surrogates for clinically meaningful outcomes. Finally, with the exception of obtaining disease prevalence rates from external sources to determine eligibility of the DRD for inclusion in the study, analyses were solely based on information reported in the recommendation documents available on the CADTH website. It was not possible to determine the extent to which these documents provided an accurate reflection of the full deliberative process that took place when formulating these recommendations.

## Conclusion

Whether a new submission is for a cancer drug or for a non-cancer drug does not appear to affect its likelihood of receiving a positive reimbursement recommendation. Safety and clinical effectiveness, not costs, appear to be the key drivers of the type of reimbursement recommendation.

## Data Availability

The datasets used and analysed during the current study are available from the corresponding author on reasonable request.

## Abbreviations

CADTH: Canadian Agency for Drugs and Technologies in Health
CDR: Common Drug Review
DRDs: Drugs for rare diseases
FDA: Food and Drug Administration
ICER: Incremental cost-effectiveness ratio
iJODR: Interim Joint Oncology Drug Review
NA: Not applicable
pCODR: Pan-Canadian Oncology Drug Review
PRO: Patient reported outcomes
RCT: Randomized controlled trial
